# Non-typeable Haemophilus influenzae Osteomyelitis and Discitis of the Cervical Vertebrae in an Elderly Adult: A Case Report and Literature Review

**DOI:** 10.7759/cureus.39155

**Published:** 2023-05-17

**Authors:** Jing Hong Fong, Shalisha Joy Menon, Gareth Iestyn Robert Jones, Hettiyadura Apsara Surangani Mendis Karunaratne

**Affiliations:** 1 Department of General Medicine, North West Regional Hospital, Burnie, AUS; 2 Department of Medical Oncology, Albury Wodonga Health, Albury, AUS; 3 Department of Medical Oncology/General Medicine, Royal Hobart Hospital, Hobart, AUS

**Keywords:** neck pain, upper respiratory tract infections, vertebral discitis, osteomyelitis, haemophilus influenzae

## Abstract

While *Haemophilus influenzae* type B (Hib) is well described in the literature to cause osteomyelitis, non-typeable *H. influenzae* has not. In areas where vaccination is routine, the prevalence of Hib has declined, whereas, in contrast, the prevalence of non-typeable *H. influenza *has increased. Generally, the non-typeable strains are less invasive but can access the vascular system by transmural migration through epithelial tight junctions or by an independent intercellular mechanism. Herein, we described a case of a 79-year-old man with the first case of non-typeable *H. influenzae* causing cervical osteomyelitis with associated bacteremia in an elderly adult.

## Introduction

Vertebral osteomyelitis is most commonly a single pathogen infection of hematogenous spread that accounts for around 3-5% of all osteomyelitis cases [1]. The majority of reported cases are caused by *Staphylococcus aureus *(42-58%) and other Gram-positive bacteria [2].

*Haemophilus influenzae* including *H. influenzae* serotype b (Hib), is a common bacterium well known to cause ear, throat, lung, and brain infections in children and was a common cause of bone and joint infection in children in the pre-vaccination era [3]. Although there are still reported cases of Hib in children, it is an extremely rare cause of osteomyelitis in adults, especially vertebral osteomyelitis with only eight other cases reported in the literature [4-12]. Among these only one case was reported to be caused by a non-typeable strain of Hib and no cases reported isolated involvement of the intervertebral disc.

We report a case of a 79-year-old male who was diagnosed with vertebral osteomyelitis secondary to non-typeable Hib in the setting of a preceding upper respiratory tract infection, who was successfully treated with antimicrobial therapy.

## Case presentation

**Figure 1:**
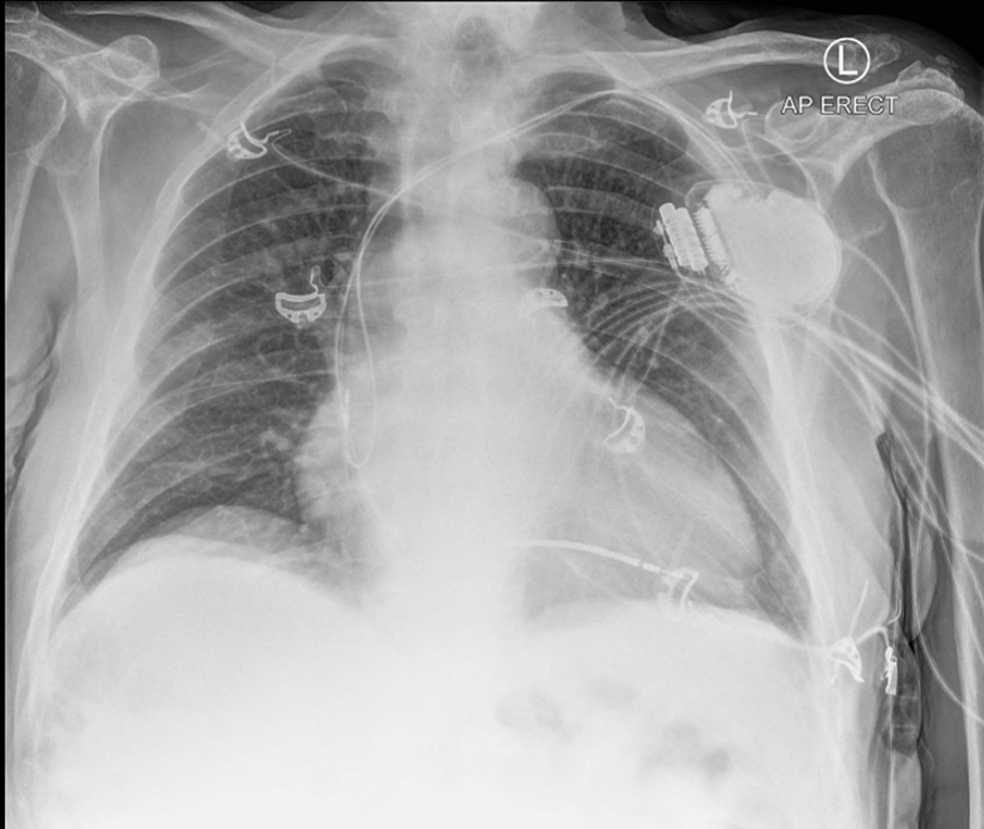
Chest-x ray taken on day 1 of presentation. Lung fields are clear with a permanent pacemaker noted at the left side of the chest.

A 79-year-old gentleman presented to the emergency department, with a several days history of acute onset of anterior neck pain with subjective fevers in a context of a recent history of presumed pharyngitis. He described severe neck pain upon waking, characterized by a sharp stabbing sensation, worse on movement, and radiating down his shoulders bilaterally. There were no overt coryzal symptoms, sinusitis, or stigmata of meningitis. His previous medical history was significant for a permanent pacemaker insertion, ischemic cardiomyopathy, dyslipidemia, and hypertension (Figure 1).

On presentation to the emergency department, his vital signs included T37.5°C (tympanic), heart rate of 110/70, pulse of 110 beats per minute, oxygen saturation of 98% on room air, and respiratory rate of 16 breaths per minute. The pharynx was erythematous but without clear exudate, and there was no palpable cervical adenopathy identified. There was tenderness over the cervical spine - specifically over C5/6, with a painful range of motion (particularly flexion/extension), and there were no neurological deficits elicited. His blood investigations showed mild leucocytosis with significantly elevated C-reactive protein (Table 1).

**Table 1:** Blood results from day 1 to day 3. MCV: mean corpuscular volume; WCC: white cell count; eGFR: estimated glomerular filtration rate; TSH: thyroid-stimulating hormone

Investigation (unit)	Day of admission		Normal range
	1	3	
Hemoglobin (g/L)	140	144	130-175
Hematocrit	0.41	0.43	0.38-0.50
MCV (fL)	89	92	80-100
WCC (nL)	11.0	10.0	4.0-11.0
Neutrophils (nL)	9.9	7.0	2.0-7.5
Lymphocytes (nL)	0.5	1.6	1.0-4.0
Monocytes (nL)	0.5	1.4	0.2-1.0
Eosinophils (nL)	<0.1	<0.1	<0.5
Basophils (nL)	<0.1	<0.1	<0.3
Platelets (nL)	150	169	150-400
Sodium (mmol/L)	140	140	135-145
Potassium (mmol/L)	4.0	4.0	4.5-5.5
Chloride (mmol/L)	106	104	95-110
Urea (mmol/L)	13.0	10.0	3.5-9.5
Creatinine (umol/L)	116	97	60-115
eGFR (mL/min/1.73 m^2^)	51	64	>89
Bicarbonate (mmol/L)	21	24	20-32
Albumin (g/L)	34	25	34-45
Total calcium (mmol/L)	2.13	2.12	2.15-2.55
Corrected calcium (mmol/L)	2.25	2.42	2.15-2.55
Phosphate (mmol/L)	0.59	0.71	0.8-1.5
Magnesium (mmol/L)	0.63	0.75	0.7-1.05
C-reactive protein (mg/L)	215	314	<5
ESR (mm/h)	14	-	0-15
TSH (mU/L)	2.2	-	0.5-5.0
Vitamin B12 (pmol/L)	176	-	200-700
Active B12 (pmol/L)	57	-	>35
Serum folate (nmol/L)	5.6	-	>7.9
25-OH vitamin D (nmol/L)	28	-	>49

**Figure 2:**
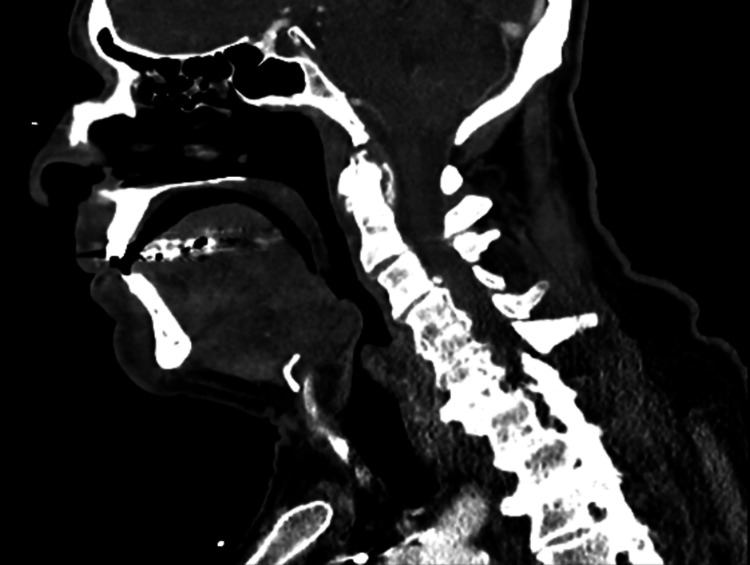
CT scan of head and neck taken on day 1 of presentation. Degenerative changes were noted throughout the cervical spine with facet hypertrophy and multilevel foraminal narrowing bilaterally.

A computerized tomography (CT) scan on the soft tissues of the neck region with intravenous (IV) contrast was organized on the day of presentation revealing degenerative changes throughout the cervical spine with facet hypertrophy, and multilevel foraminal narrowing bilaterally (Figure 2). Of note, there was no significant bone destruction/erosive changes, pre-vertebral tissue swelling, or lymphadenopathy. While mild mucosal thickening was seen in the ethmoidal cells, there was no evidence of sinusitis. However, osteomyelitis discitis cannot be excluded on CT, and given the history of fevers, and raised inflammatory markers, magnetic resonance imaging (MRI) was recommended by the radiologist for further evaluation.

**Figure 3:**
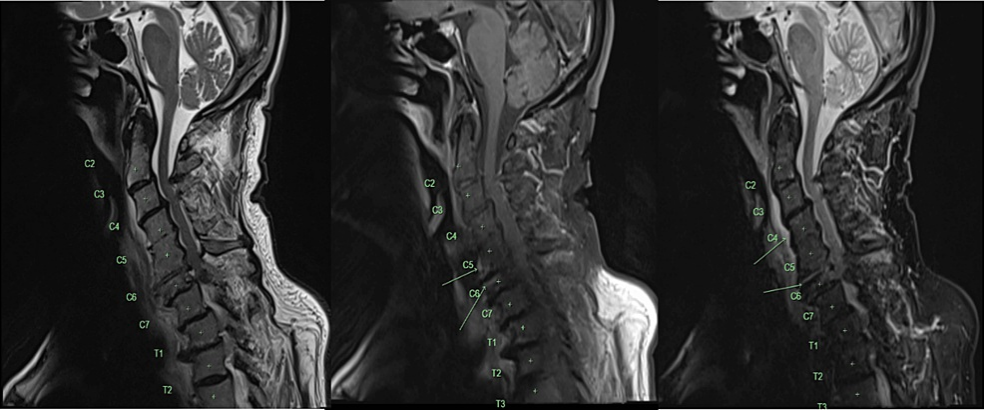
MRI of head and neck. Findings showed the presence of extensive degenerative change, most pronounced at C5/C6 where there is obliteration of the disc space and extensive peripheral osteophytosis.

The patient was then admitted into the ward under the general medical unit under the provisional diagnosis of osteomyelitis and spondylodiscitis. He was commenced on IV flucloxacillin 2 g every 6 h. MRI of the cervical spine was done on the following day (day 2) (Figure 3). Findings showed the presence of extensive degenerative change, most pronounced at C5/C6 where there is obliteration of the disc space and extensive peripheral osteophytosis. Residual disc space at this level is a diffusely high fluid signal. Adjacent anterior endplates were hyperenhanced although without notable edema. Pre-vertebral inflammatory change is present, with edema and mild hyperenhancement extending along the anterior vertebral column. No pre-vertebral collection is present.

Moreover, there is also severe multilevel canal stenosis, most pronounced at C5/C6, with complete cerebral spinal fluid (CSF) effacement but normal preserved cord signal. Multilevel severe foraminal stenoses are present. Radiologist noted that in the given clinical context, an early osteomyelitis discitis is the leading concern, although features are not completely typical, and the advanced degeneration alone could result in the vertebral signal change.

On day 3, the initial result of blood cultures came back and was revealed to be growing a Gram-negative bacillus. On day 4, the result came through. It was noted that *H. influenzae *had been detected from both aerobic and anaerobic bottles. The organism is tested to be sensitive to ampicillin/amoxicillin and ceftriaxone. Further testing was done, and the laboratory found out that the organism was serotyped negative for type B *H. influenzae*. A final diagnosis of non-typeable *H. influenzae *osteomyelitis and discitis of the C5/6 was made.

His case was discussed with the infectious diseases unit and was changed to IV ceftriaxone 2 g BD. A transthoracic echocardiogram (TTE) was organized to rule out endocarditis, which is a potential complication. Transesophageal echocardiogram (TEE) is the gold standard of diagnosis of infective endocarditis and not TTE. However, our patient did not have evidence of endocarditis and he was not keen on doing TEE. The TTE did not reveal valvular dysfunction or overt vegetation.

The patient showed significant clinical and biochemical improvement throughout his admission. Blood cultures sent on day 4 and day 6 showed no growth after two days of incubation, and he was able to be discharged on day 9. He was given a plan to complete a total of six weeks of IV ceftriaxone daily and follow-up with the infectious disease outpatient clinic upon completion of antimicrobial therapy (Table 2).

**Table 2:** Blood investigation results after the commencement of IV cefazolin from day 5 to day 8. MCV: mean corpuscular volume; eGFR: estimated glomerular filtration rate; HCT: hematocrit; WCC: white cell count; ALP: alkaline phosphatase; ALT: alanine transaminase

Investigation (unit)	Day of admission				Normal range
	5	6	7	8	
Hemoglobin (g/L)	133	136	135	128	130-175
HCT	0.39	0.39	0.39	0.38	0.38-0.50
MCV (fL)	90	90	88	92	80-100
WCC (nL)	7.4	9.2	8.2	8.7	4.0-11.0
Neutrophils (nL)	4.4	5.8	5.0	6.0	2.0-7.5
Lymphocytes (nL)	1.9	2.3	2.2	1.9	1.0-4.0
Monocytes (nL)	1.0	0.9	0.8	0.7	0.2-1.0
Eosinophils (nL)	0.1	0.2	0.2	0.1	<0.5
Basophils (nL)	<0.1	<0.1	<0.1	<0.1	<0.3
Platelets (nL)	186	242	270	315	150-400
Sodium (mmol/L)	140	139	141	141	135-145
Potassium (mmol/L)	4.0	4.1	4.5	4.5	4.5-5.5
Chloride (mmol/L)	106	105	103	105	95-110
Urea (mmol/L)	6.4	5.6	4.9	5.7	3.5-9.5
Creatinine (umol/L)	77	78	76	81	60-115
eGFR (mL/min/1.73 m^2^)	82	82	83	79	>89
Bicarbonate (mmol/L)	22	22	26	26	20-32
Total bilirubin (umol/L)	-	10	12	9	4-20
ALP (U/L)	-	94	89	90	35-110
Gamma GT (U/L)	-	121	118	114	5-50
ALT (U/L)	-	25	25	23	5-40
Total protein (g/L)	-	58	58	61	63-80
Globulin (g/L)	-	34	34	35	26-41
Albumin (g/L)	23	24	24	26	34-45
Total calcium (mmol/L)	2.06	2.07	2.11	2.13	2.15-2.55
Corrected calcium (mmol/L)	2.40	2.39	2.43	2.41	2.15-2.55
Phosphate (mmol/L)	0.94	1.04	1.15	0.96	0.8-1.5
Magnesium (mmol/L)	0.61	0.63	0.66	0.70	0.7-1.05
C-reactive protein (mg/L)	185	126	109	78	<5

**Figure 4:**
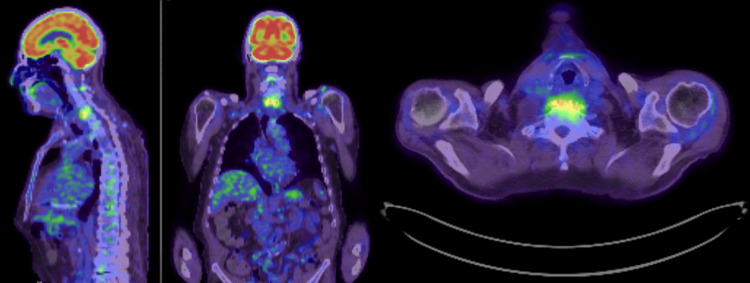
The PET scan showed curvilinear activity pertaining to C5/C6 endplates and vertebral body, compatible with the known discitis/osteomyelitis.

On day 21, he was admitted for three days of keto diet to prepare himself for a 245 MBq fludeoxyglucose F 18 injection (FDG) positron emission tomography (PET) scan scheduled on day 24. The uptake time was 60 minutes with a blood sugar level of 3.8 mmol/L. The PET scan showed curvilinear activity pertaining to C5/C6 endplates and vertebral body, compatible with the known discitis/osteomyelitis (Figure 4). On clinic follow-up, the patient had recovered completely and did not have active complaints.

## Discussion

**Figure 5:**
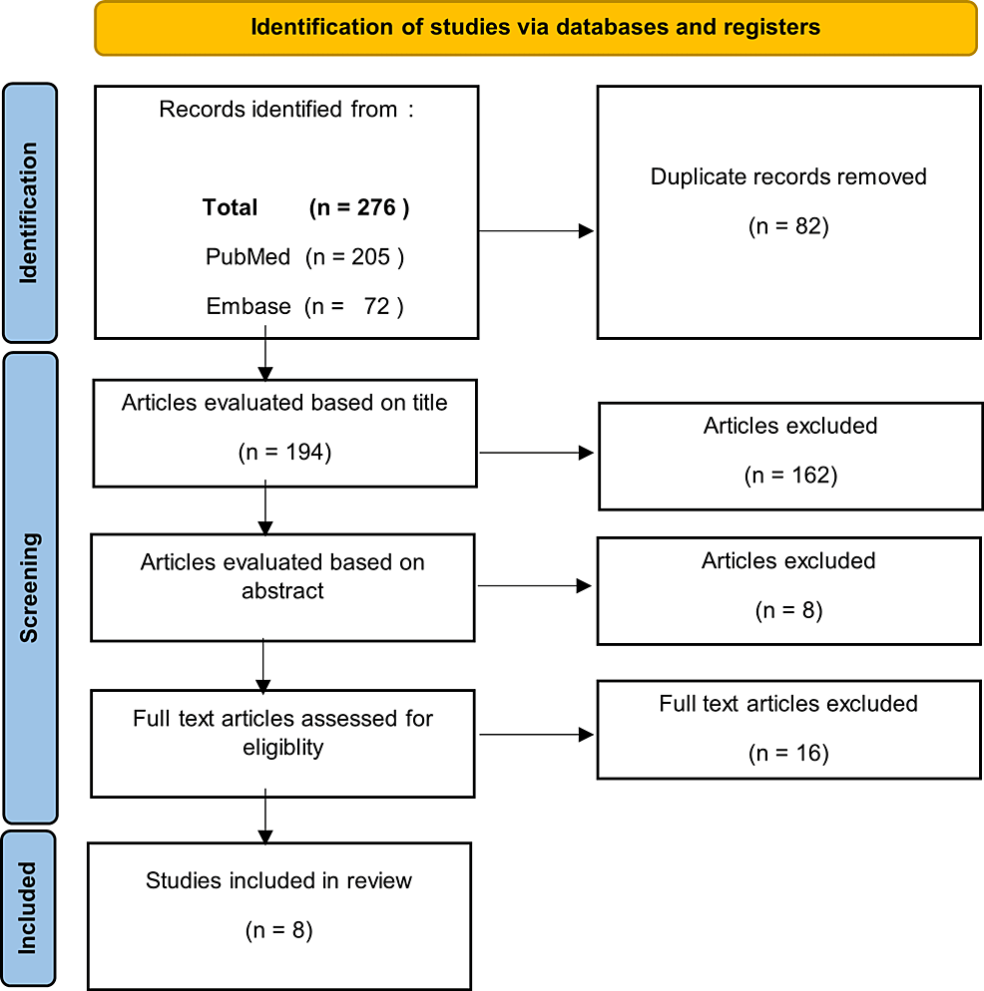
The literature search was done systematically as per the PRISMA chart. PRISMA: Preferred Reporting Items for Systematic Reviews and Meta-Analyses

Vertebral osteomyelitis caused by *H. influenzae *is rare. A systematic review was performed in accordance with the PRISMA chart and yielded 10 reported cases from 1978 to 2012 [4-12]. Articles for this review were identified through databases such as PubMed, Embase, and Google Scholar without start date restrictions. Keywords such as “osteomyelitis” OR “spondylodiscitis” AND “*H. influenzae*” were used. All results from the searches and their respective cited references were reviewed. Only articles published in English were included (Figure 5).

Among them, five cases (50%) were caused by type B *H. influenzae*. The site of involvement in nine of the cases (90%) was found in the lumbar region and in one case in the thoracic region. We here report the first case of vertebral osteomyelitis caused by a non-typeable *H. influenzae *(NTHi) that was found in the cervical region.

*H. influenzae *is a small (1×0.3 µ), non-motile, Gram-negative rod that was first reported by Pfeiffer in 1892. *H. influenzae *is pleomorphic and commonly colonizes and infects the human respiratory tract. The species is divided into typeable (encapsulated) and non-typeable (unencapsulated) strains. Non-typeable strains are classified into biotypes based on the presence or absence of indole, urease, and ornithine decarboxylase. Non-typeable strains are genetically heterogenous and differ in their pathogenic potential [13,14].

**Figure 6:**
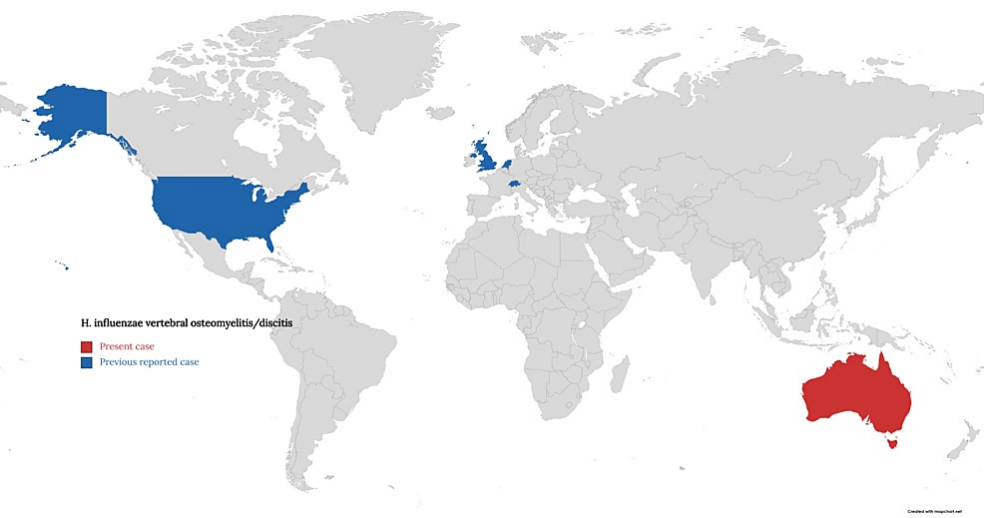
World map highlighting the reported cases of vertebral osteomyelitis caused by H. influenzae. The image is created by the author of this study.

*H. influenzae *serotype b (Hib) is the most virulent strain among the typeable strains. Hib is a significant cause of meningitis and epiglottitis in children as well as pneumonia in adults in regions of the world where Hib immunization is not widely practiced (Figure 6). On the other hand, the prevalence of Hib has decreased in areas with routine Hib vaccination whereas the prevalence of non-typeable *H. influenzae *has increased [15]. A study conducted in 2015 showed that invasive NTHi disease is increasing worldwide and is also the commonest pathogen causing pneumonia in patients with chronic obstructive pulmonary disease (COPD) and osteomyelitis in children [16].

NTHi is known to cause a lot of non-invasive infections [14]. For example, sinusitis, otitis media, conjunctivitis, exacerbations of COPD, and non-bacteremic pneumonia [17]. NTHi can cause invasive infections as well, as it is one of their critical pathological features. Invasive disease refers to infection that extends beyond the respiratory tract. NTHi invades the vascular system by transmural migration via epithelial tight junctions or by an independent intercellular mechanism [14]. However, invasive infections are mostly seen in neonates, presenting as sepsis and meningitis around 1.6-4.9 in every 100 live births [18]. Vertebral osteomyelitis, as presented in this case, is considered an invasive disease. The incidence of hospitalization for vertebral osteomyelitis is on the rising trend, especially in the United States and France, which was reported to be doubled [1,19].

The patient we reported had no clear causative factor. Unlike the patient described in the study by Boulton et al. who had prior dental procedures, there were no recent surgical procedures done for our patient [12]. No significant travel history or exposure to sick contacts was reported. The only possible causative factor is his recent pharyngitis. His presentation is unusual, which was described as a sharp stabbing sensation in the neck, worse on movement, and radiating down his shoulders bilaterally.

In terms of identifying *H. influenzae *in the case of invasive infection, blood culture is still highly specific but is dependent on the quality of specimen handling. Matrix-assisted laser desorption/ionization time-of-flight (MALDI-TOF) mass spectrometry has a higher sensitivity and specificity than blood culture but is unable to differentiate between *H. influenzae *strains. PCR-based assays would be the best method of identification because PCR-based assays have high sensitivity, high specificity, and rapid turnaround time. Besides that, PCR-based assays can be of specimens from patients who had their empirical antibiotic commenced prior [20].

The most sensitive imaging technique for vertebral osteomyelitis is MRI, showing decreased signal intensity in the vertebral bodies and disc and loss of endplate definition. If MRI is unavailable, a CT scan would be a good second choice. In cases where MRI is contraindicated, such as in claustrophobic patients or due to the presence of implants or metal devices, radionuclide scanning may be preferred instead [19]. Similar to this study, four previous case reports commencing their patients on empirical antibiotics from the penicillin family [5,8,11,12]. However, the majority switched to IV ceftriaxone following organism identification (Table 3).

**Table 3:** Clinical characteristics of 16 adult patients with H. influenzae vertebral osteomyelitis/discitis. CSF: cerebrospinal fluid; CVD: cardiovascular disease; DM: diabetes mellitus; ESRF: end-stage renal failure; HPT: hypertension; L: lumbar vertebrae; LBP: low back pain; NS: not stated; RTI: respiratory tract infection; T: thoracic vertebrae; TMP-SMZ: trimethoprim-sulfamethoxazole; UTI: urinary tract infection

Reference			Age/sex	Organism	Co-morbid	Site of involvement	Clinical manifestations	Antibiotic regimen/duration	Outcome/length of follow-up
Ref no.	Country	Year							
[4]	USA	1978	52/F	Type b	None	L3-4	LBP 3 months	IV cephalothin/2 weeks IM cefazolin/4 weeks	Improved/4 months
[5]	Switzerland	1984	72/M	Type b	None	L3-4	LBP 9 months, decreased deep tendon reflexes	IV ceftriaxone; PO TMP-SMZ	Recovered/NS
[5]	Switzerland	1984	36/F	Type c	UTIs	L2-3	LBP 4 months, fever	IV ampicillin/3 weeks; IV ceftriaxone/1 week; PO doxycycline	Recovered/ NS
[6]	USA	1987	39/M	Type b	RTI	L3	LBP 3 months, weight loss	IV ceftizoxime/6 weeks	Recovered/ 12 months
[6]	USA	1987	59/F	Type b	RTI	L3	LBP 4 months	IV ceftriaxone/6 weeks	Recovered/ 18 months
[7]	USA	1989	66/M	Type b	DM	T9-T10	LBP 4 months, weight loss	IV ceftriaxone/6 weeks	Recovered/ 12 months
[8]	Netherlands	1991	66/M	NS	RTI	L2-3	LBP 2 months	IV amoxicillin/2 weeks, PO amoxicillin/6 weeks	Recovered/ 12 months
[9]	USA	1997	59/F	Non-typeable	DM	L4-S1	LBP 2 months, fever, weight loss	PO ciprofloxacin/3 weeks, PO lomefloxacin/7 weeks, IV ceftriaxone/2 weeks	Slowly improved/ NS
[11]	Netherlands	2008	66/M	Non-typeable	CVD, DM, ESRF	L2-3	Severe LBP, fever	IV amoxicillin/7 weeks, IV ceftriaxone/6 months	Recovered/4 months
[12]	United Kingdom	2012	67/M	Non-typeable	Gastritis, HPT, nodular prurigo	L4-5	LBP, painful right shoulder, fever, rigors.	IV co-amoxiclav/NS, IV ceftriaxone/6 weeks PO antibiotics/6 weeks	Recovered/NS
This case	Australia	2022	79/M	Non-typeable	Permanent cardiac pacemaker, IHD, HPT	C5-6	Anterior neck pain, worse on movement, fever, painful shoulders bilaterally	IV flucloxacillin/2 days, IV ceftriaxone/6 weeks	Recovered/2 months

## Conclusions

Although uncommon, vertebral osteomyelitis caused by non-typeable *H. influenzae* is manageable and treatable. The utilization of imaging and blood culture is crucial to reach an accurate diagnosis. If the clinical circumstances allow it, tissue from the disc should be sampled before beginning any empirical treatment. This is the first reported case where the infection site is the cervical region. Our case contributes to the body of existing literature on a new potential cause of cervical osteomyelitis for the awareness of clinicians.
